# Safety Profile of Immunotherapy Combined With Antiangiogenic Therapy in Patients With Melanoma: Analysis of Three Clinical Studies

**DOI:** 10.3389/fphar.2021.747416

**Published:** 2021-11-09

**Authors:** Hui Tian, Xuan Wang, Bin Lian, Xieqiao Yan, Lu Si, Zhihong Chi, Xinan Sheng, Yan Kong, Lili Mao, Xue Bai, Bixia Tang, Siming Li, Li Zhou, Chuanliang Cui, Jun Guo

**Affiliations:** Key Laboratory of Carcinogenesis and Translational Research (Ministry Education), Department of Melanoma, Peking University Cancer Hospital and Research Institute, Beijing, China

**Keywords:** combination therapy, safety profile, immunetherapy, antiangiogenic therapy, melanoma

## Abstract

**Objective:** To describe the frequency and spectrum of treatment-related adverse events (TRAEs) of immunotherapy combined with antiangiogenic therapy in patients with melanoma.

**Methods:** This retrospective cohort study included three clinical trials on patients with stage III/IV melanoma treated with anti–PD 1 and antiangiogenic therapy.

**Results:** We analyzed data from 72 patients with a median follow-up time of 25.9 months (95% CI, 9.1–42.7 m). The median treatment duration was 7.5 months (range, 0.7–42.8 m), and the median of treatment cycles was 11.0 (range, 1–90). Most patients (70 of 72 or 97.2%) experienced TRAEs (mostly grades 1 or 2). No drug-related deaths were reported. Most TRAEs were hepatic (75%), endocrine (72.2%), skin (65.3%), and gastrointestinal tract (59.7%) manifestations, followed by myelosuppression (55.6%), renal dysfunction (55.6%), and dyslipidaemia (54.2%). The adverse event (AE) spectra were similar between regimens. Using multivariate Cox proportional risk models showed that hypertension was associated with a long PFS. According to our multivariable logistic regression models, TRAEs were not associated with ORR.

**Conclusion:** We found that the prevalence of AEs was higher than that of anti–PD-1 monotherapy. Most of the AEs were mild. The AE spectra were similar to those seen after anti–PD-1 or antiangiogenic therapy monotherapy, without unexpected AEs. Immunotherapy combined with antiangiogenic therapy was well tolerated.

**Clinical Trial Registration**: ClinicalTrials.gov, identifier NCT03955354.

## Introduction

Melanoma is an aggressive malignancy with a dismal prognosis. The incidence of melanoma has increased annually (Miller et al., 2019; Miller et al., 2020). In the United States, more than 100,000 new melanoma cases have emerged, and more than 7,000 people die annually from the disease (Hodi et al., 2010). Immunotherapy [including antibodies against cytotoxic T-lymphocyte–associated antigen 4 (CTLA-4), programmed cell death protein 1 (PD-1), and programmed death ligand-1 (PD-L1)] is the most important development in cancer therapy of the past 10 years. Some prospective studies have shown that immunology greatly improved the clinical outcomes of patients with cutaneous advanced melanoma (Hodi et al., 2010; Wolchok et al., 2015; Robert et al., 2019). The National Comprehensive Cancer Network (NCCN) recommends anti–PD-1 monotherapy as the standard treatment for advanced cutaneous melanoma in its clinical practice guidelines. Acral and mucosal melanomas are the two most common melanoma subtypes in Asia, comprising nearly two-thirds of all melanomas (Chi et al., 2011). Acral and mucosal melanomas can metastasize earlier in the disease process than cutaneous melanomas due to their distinctive biological features (Carvajal et al., 2012). Unfortunately, anti–PD-1 as monotherapy does not provide the same survival benefits in patients with acral and mucosal melanoma as it does in patients with cutaneous melanomas (Tang et al., 2020). However, anti–PD-1 combined with antiangiogenic therapy has brought new hope to these patients. A phase Ib trial showed a survival benefit from combination therapy in patients with advanced mucosal melanoma (Sheng et al., 2019). Combination therapy has also proven efficacious against a wide range of tumors, including hepatocellular carcinoma (Finn et al., 2020) and endometrial cancer (Makker et al., 2020). The adverse event (AE) spectrum of combination therapy is thought to differ from that of the anti–PD-1/-PD-L1 regimen, but the available evidence is mainly derived from a series of small prospective studies (Sheng et al., 2019). Thus, we summarized three clinical trial AEs to better describe the safety profile of anti–PD-1 therapy combined with antiangiogenic therapy.

## Materials and Methods

### Study Design and Patients

All data in this study are derived from patients treated with at least one cycle of anti–PD-1 plus antiangiogenic therapy enrolled in one of the following clinical trials performed at the Peking University Cancer Hospital: (1) a phase IB nonrandomized, open-label, dose-finding trial on patients with metastatic mucosal melanoma (*n* = 33, patients received toripalimab plus axitinib, ClinicalTrials.gov identifier: NCT03086174); (2) a phase II randomized, open-label, multicenter trial on first-line treatment of patients with metastatic mucosal melanoma (combination therapy cohort n = 9, patients received toripalimab plus axitinib, ClinicalTrials.gov identifier: NCT03955354); and (3) a phase II study on first-line treatment of patients with unresectable stage III or IV acral melanoma (*n* = 30, patients received camrelizumab plus apatinib, ClinicalTrials.gov identifier: NCT03955354).

Investigators evaluated the responses using RECIST (version 1.1.) with AEs graded according to the National Cancer Institute Common Terminology Criteria for Adverse Events version 4.03 (CTCAE v4.03). The drugs involved in the trials were the following: toripalimab and camrelizumab, both humanized anti–PD1 IgG4 monoclonal antibodies; apatinib, a small molecule tyrosine kinase inhibitor selectively inhibiting the vascular endothelial growth factor receptor 2 (VEGFR-2); and axitinib, a small molecule tyrosine kinase inhibitor of VEGFR 1-3, c-KIT, and PDGFR.

### Data Collection

We collected data from three clinical trials, including two phase II and one phase IB trial. Patients were treated until disease progression or unacceptable toxicity or for up to 2 years and then switched to anti–PD-1 monotherapy maintenance. We collected the following clinical data: patient demographics (age, sex, genetic mutation status, etc.), pathological tumor type, treatment (therapeutic regimen, treatment duration, and number of completed treatment cycles), and outcomes. Safety profile data were collected from the date of the first dose of anti–PD-1 plus antiangiogenic therapy to withdrawal from the trial. In addition, we collected the following safety profile data: AE grades, AE types, onset date, AE resolution data, and AE outcomes. The date of AE onset was defined as the time in which abnormal laboratory testing or associated symptoms occurred for the first time. The AE resolution date was defined as the date of complete resolution or improvement to the baseline grade. AEs’ time to onset was defined as the time from treatment start to the date of AE onset. AEs’ time to resolution was defined as the longest time from the date of AE onset to resolution.

We divided AEs into several different categories (according to the affected system) including hepatic, endocrine dysfunctions, dermatologic toxicity, and others. PFS was defined as the time (in months) from the date of the first dose of combined therapy to disease progression, and OS was defined as the time (in months) from the date of the first dose of combined therapy to death from any cause.

### Statistical Analysis

We used frequencies and percentages to describe categorical variables, and we used medians and ranges to describe continuous variables. The 95% confidence intervals (CIs) of the objective response rate (ORR) and the disease control rate (DCR) were estimated using the Clopper–Pearson method. We plotted PFS survival curves using the Kaplan–Meier method and compared variables between groups using the log-rank test. We performed univariate and multivariate logistic regression models to estimate the risk ratio (RR) and its associated 95% CI to describe the risk for AEs on the ORR (adjusted for age, gender, race, ECOG, pathological type of tumor, and stage). Moreover, we used univariate and multivariate Cox proportional hazards regression models to identify the factors associated with PFS and to calculate the hazard ratio (HR) and its associated 95% CI. In all, we assessed the following factors: age, sex, genetic mutation status, pathological type of tumor, level of LDH, ECOG, stage, and AEs (hypertension, renal toxicity, vitiligo, endocrine dysfunction, and others). All analyses were performed using SPSS, version 23 (IBM, Armonk, NY, United States) and GraphPad PRISM, version Prism 6 (GraphPad Software, LLC). All tests were two-sided, and we considered *p*-values < 0.05 as statistically significant.

## Results

### Baseline Clinical Characteristics

Table 1 lists patient demographics. A total of 72 patients were included, of whom 30 (41.7%) received camrelizumab plus apatinib and 42 (58.3%) received toripalimab plus axitinib. The median age of patients was 56.0 (IQR, 49.0–63.8), and there were more women than men (54.2 vs. 45.8%). Most patients (*n* = 54) had stage IV advanced melanoma. We found that 58.3% of patients presented mucosal melanoma and 30 (41.7%) had acral melanoma. Eighteen patients (25%) harbored genetic mutations, most of them had RAS mutations (12.5%; NRAS n = 8, KRAS *n* = 1), and the next most common genetic mutations were of the BRAF v600 type (BRAFv600e n = 5, BRAFv600k *n* = 1). Two-thirds of patients had an Eastern Cooperative Oncology Group (ECOG) performance status of 0, and 26 (36.1%) patients had elevated serum LDH levels. Most patients had not received systemic therapy previously. The median follow-up time was 25.9 months (95% CI, 9.1–42.7 months). The median treatment duration was 7.5 months (range, 0.7–42.8 months), and the median treatment cycles were 11.0 (range, 1–90). The median immunotherapeutic drug doses were 11.0 (range 1–90).

**TABLE 1:** Patients’ demographics.

Characteristics	Specifications	*n* = 72
Age, years	Median (IQR)	56.0 (49.0–63.8)
Sex, n (%)	Male	33 (45.8)
	Female	39 (54.2)
Pathological type, n (%)	Mucosal	42 (58.3)
	Acral	30 (41.7)
Mutation status, n (%)	BRAF	6 (8.3)
	RAS	9 (12.5)
	KIT	3 (4.2)
	BRAF/RAS/KIT wild type	31 (43.1)
	UK	23 (31.9)
Therapeutic regimen, n (%)	Camrelizumab + apatinib	30 (41.7)
	Toripalimab + axitinib	42 (58.3)
LDH, n (%)	≤ULN	46 (63.9)
	>ULN	26 (36.1)
ECOG, n (%)	0	48 (66.7)
	1	24 (33.3)
AJCC stage, n (%)	III	18 (25.0)
	IV, M1a	20 (27.8)
	IV, M1b	21 (29.2)
	IV, M1c	13 (18.0)
Prior systemic therapy, n (%)	No	62 (86.1)
	Yes	10 (13.9)
Treatment duration, months	Median	7.5 (0.7–42.8)
Treatment circles	Median (range)	11.0 (1–90)

AJCC, American Joint Committee on Cancer; ECOG, Eastern Cooperative Oncology Group; LDH, lactic dehydrogenase.

### Overall Analysis

Table 2 lists the TRAEs suffered by 70 of 72 (97.2%) patients. Most adverse events (AEs) were grade 1 or 2. Most TRAEs were hepatic (75%) with abnormal liver function test results and hepatitis. Other common TRAEs included endocrine (72.2%), skin (65.3%), and gastrointestinal tract (59.7%) dysfunctions, myelosuppression (55.6%), renal abnormalities (55.6%), and dyslipidaemia (54.2%) in more than half of the patients ( Supplementary Table S1 lists all AEs occurring in >10% of patients). All cardiac or neurological TRAEs were grade 1 or 2 and included arrhythmia (20.8%), abnormal ECG (16.7%), and headache (12.5%). Almost half of the patients (*n* = 35) experienced grade 3 or higher AEs. Grade 4 AEs occurred in five patients and included increased alanine aminotransferase (ALT; *n* = 1), increased aspartate aminotransferase (AST; *n* = 1), increased amylase (*n* = 1), high triglyceride levels (*n* = 1), and increased creatine kinase (*n* = 1). All of the AEs improved to grade 3, four cases after treatment interruption and one case after symptomatic relief treatment. Treatment interruptions occurred in 32 patients (48.6%), of whom four patients discontinued the treatment permanently. We found no treatment-related deaths. Serious AEs occurred in nine patients and included myositis (*n* = 1), hepatitis (*n* = 2), increased ALT and AST (*n* = 1), oesophageal fistula (*n* = 1), hyperbilirubinemia (*n* = 1), rash (*n* = 1), pneumonitis (*n* = 1), and proteinuria (*n* = 1).

**TABLE 2:** Treatment-related adverse events (TRAEs).

n (%)	Total *n* = 72	PD-1+APA *n* = 30	PD-1+AXI *n* = 3042
TRAEs	70 (97.2)	30 (100.0)	40 (95.2)
Any grade	70 (97.2)	30 (100.0)	40 (95.2)
Grades 3–5	35 (48.6)	17 (56.7)	18 (42.9)
G3	35 (48.6)	17 (56.7)	18 (42.9)
G4	5 (6.9)	3 (10.0)	2 (4.8)
G5	0	0	0
Serious AEs	9 (12.5)	4 (13.3)	5 (11.9)
TRAEs leading to discontinuation	32 (44.4)	14 (46.7)	18 (42.9)
TRAEs leading to death	0	0	0

**FIGURE 1:**
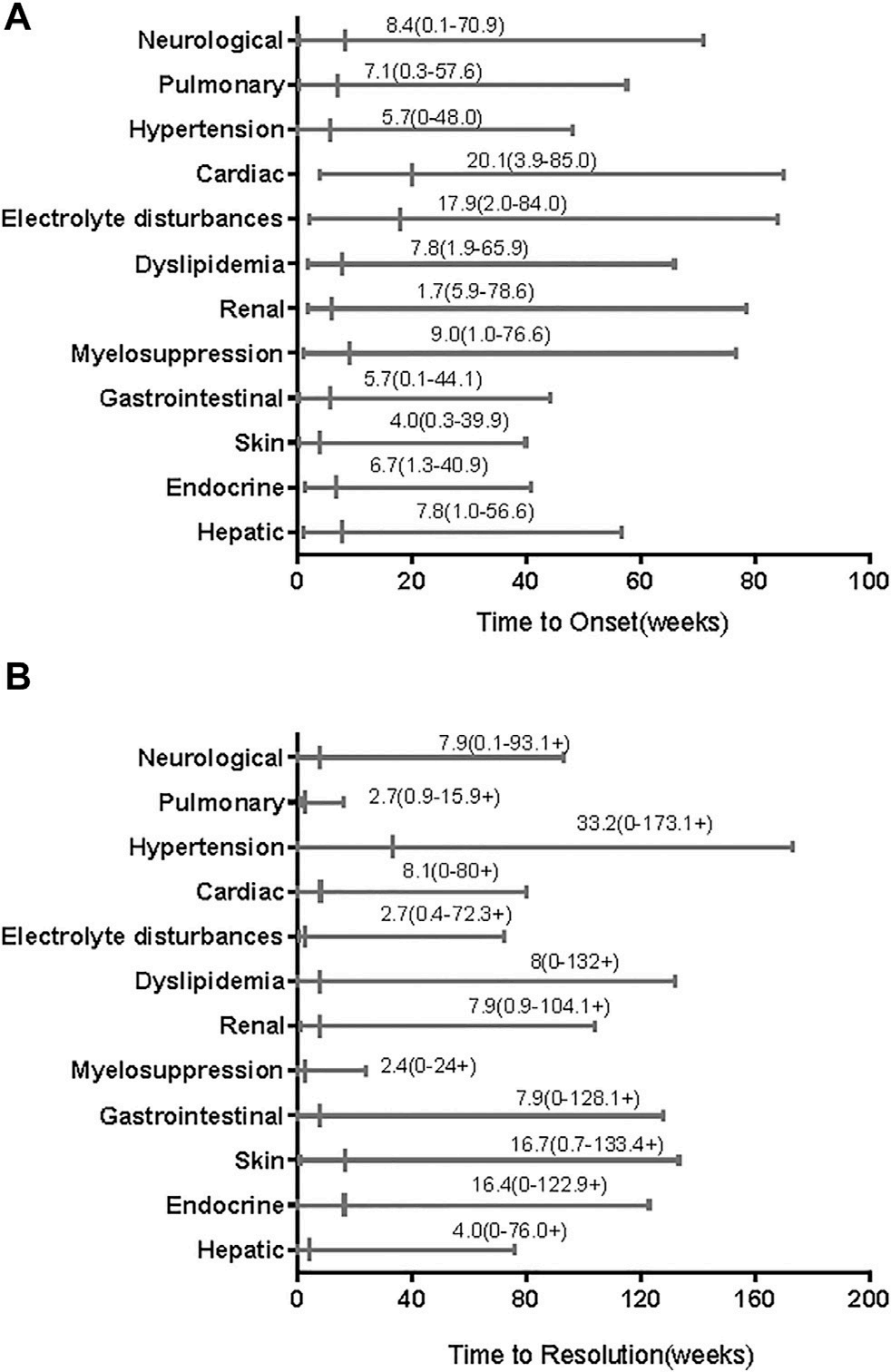
Time to onset and resolution of treatment-related adverse events (TRAEs) of any grade. The small vertical lines represent medians and the bars indicate ranges. **(A)** Time to onset; **(B)** time to resolution. The symbols “+” in ranges indicate censored values.

Table 3 summarizes the common AEs (>15%) and some TRAEs of special interest that we found. As shown in that table, the most AEs (of all grades) of clinical symptoms were hand-foot syndrome (*n* = 31), diarrhoea (*n* = 27), rash (*n* = 22), and hypertension (*n* = 21). Furthermore, most all-grade AEs evidenced by abnormal laboratory testing results were increased ALT (*n* = 36), proteinuria (*n* = 34), hyperbilirubinemia (*n* = 32), hypothyroidism (*n* = 28), and high cholesterol (*n* = 28), followed by high triglycerides (*n* = 27), leukopenia (*n* = 26), and increased AST (*n* = 24). The most common grade ≥3 AEs were increased ALT (*n* = 12), proteinuria (*n* = 5), and high triglycerides (*n* = 5), followed by diarrhoea (*n* = 4), neutropenia (*n* = 4), and increased AST (*n* = 4). TRAEs of special interest that occurred in less than 15% of patients included vitiligo (*n* = 9), hepatitis (*n* = 4), myositis (*n* = 1), and pneumonitis (*n* = 1). The median time of all-grade TRAE onset ranged from 4 weeks for skin AEs to 20.1 weeks for cardiac AEs (Figure 1A). Most all-grade TRAEs were resolved within several weeks, but most mild AEs continued throughout the treatment (Figure 1B).

**TABLE 3:** Common adverse events (>15%) and some TRAEs of special interest patients.

Adverse event, n (%)	*n* = 72	
	Any grade	Grade 3 or 4
Clinical symptoms		
Hand-foot syndrome	31 (43.1)	0
Diarrhoea	27 (37.5)	4 (5.6)
Rash	22 (30.6)	0
Hypertension	21 (29.2)	2 (2.8)
Fatigue	19 (26.4)	2 (2.8)
Joint or muscle pain	17 (23.6)	0
Weight loss	15 (20.8)	1 (1.4)
Oral mucositis	14 (19.4)	0
Nausea	13 (18.1)	0
Abdominal pain	12 (16.7)	0
Appetite decreased	10 (13.9)	1 (1.4)
Headache	9 (12.5)	0
Vitiligo	9 (12.5)	0
Hoarseness	8 (11.1)	0
Myositis	1 (1.4)	1 (1.4)
Pneumonitis	1 (1.4)	0
Laboratory testing abnormal		
ALT increased	36 (50.0)	12 (16.7)
Proteinuria	34 (47.2)	5 (6.9)
Hyperbilirubinemia	32 (44.4)	1 (1.4)
Hypothyroidism	28 (38.9)	0
High cholesterol	28 (38.9)	0
High triglyceride	27 (37.5)	5 (6.9)
Leukopenia	26 (36.1)	1 (1.4)
AST increased	24 (33.3)	4 (5.6)
Hyperglycaemia	19 (26.4)	0
Hypokalaemia	17 (23.6)	0
Creatine kinase increased	17 (23.6)	2 (2.8)
Arrhythmia	15 (20.8)	0
Neutropenia	15 (20.8)	4 (5.6)
Uric acid increased	14 (19.4)	0
Amylase increased	12 (16.7)	0
Abnormal ECG	12 (16.7)	0
Thrombocytopenia	12 (16.7)	0
Hyperthyroidism	11 (15.3)	0
Anaemia	11 (15.3)	2 (2.8)

**FIGURE 2:**
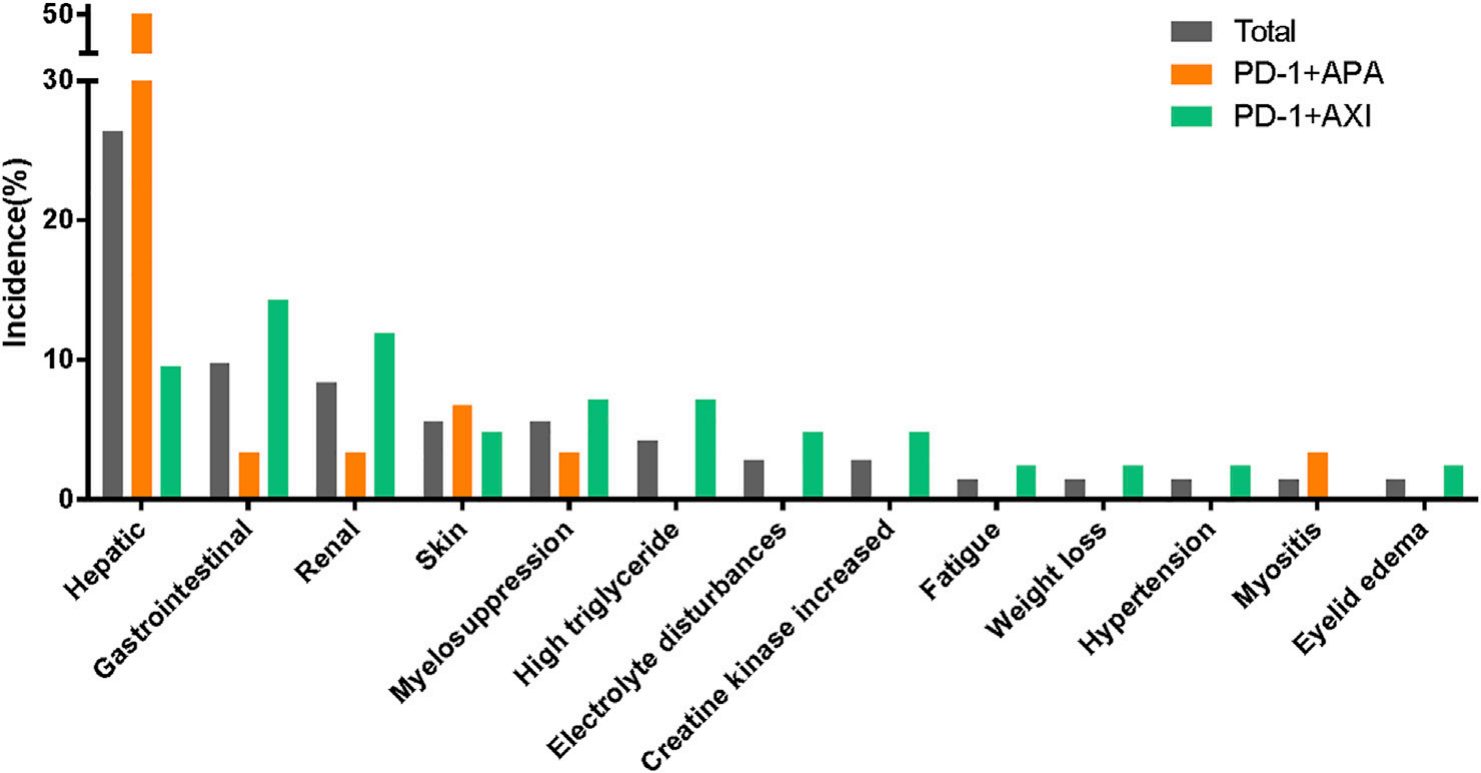
Treatment-related adverse events leading to discontinuation between different therapeutic regimens.

Treatment-related AEs leading to treatment discontinuation were reported in 32 patients; in them, the most frequent AEs were increased ALT (*n* = 9), increased AST (*n* = 6), proteinuria (*n* = 5), hepatitis (*n* = 4), diarrhoea (*n* = 3), and high triglyceride levels (*n* = 3). Please see Figure 2; Supplementary Table S2.

### Incidence of Immune-Related and Antiangiogenic Therapy–Related Adverse Events

Endocrine dysfunctions and inflammation of other organs including hepatitis, pneumonitis, myocarditis, and colitis have been associated with immunotherapy.

**FIGURE 3:**
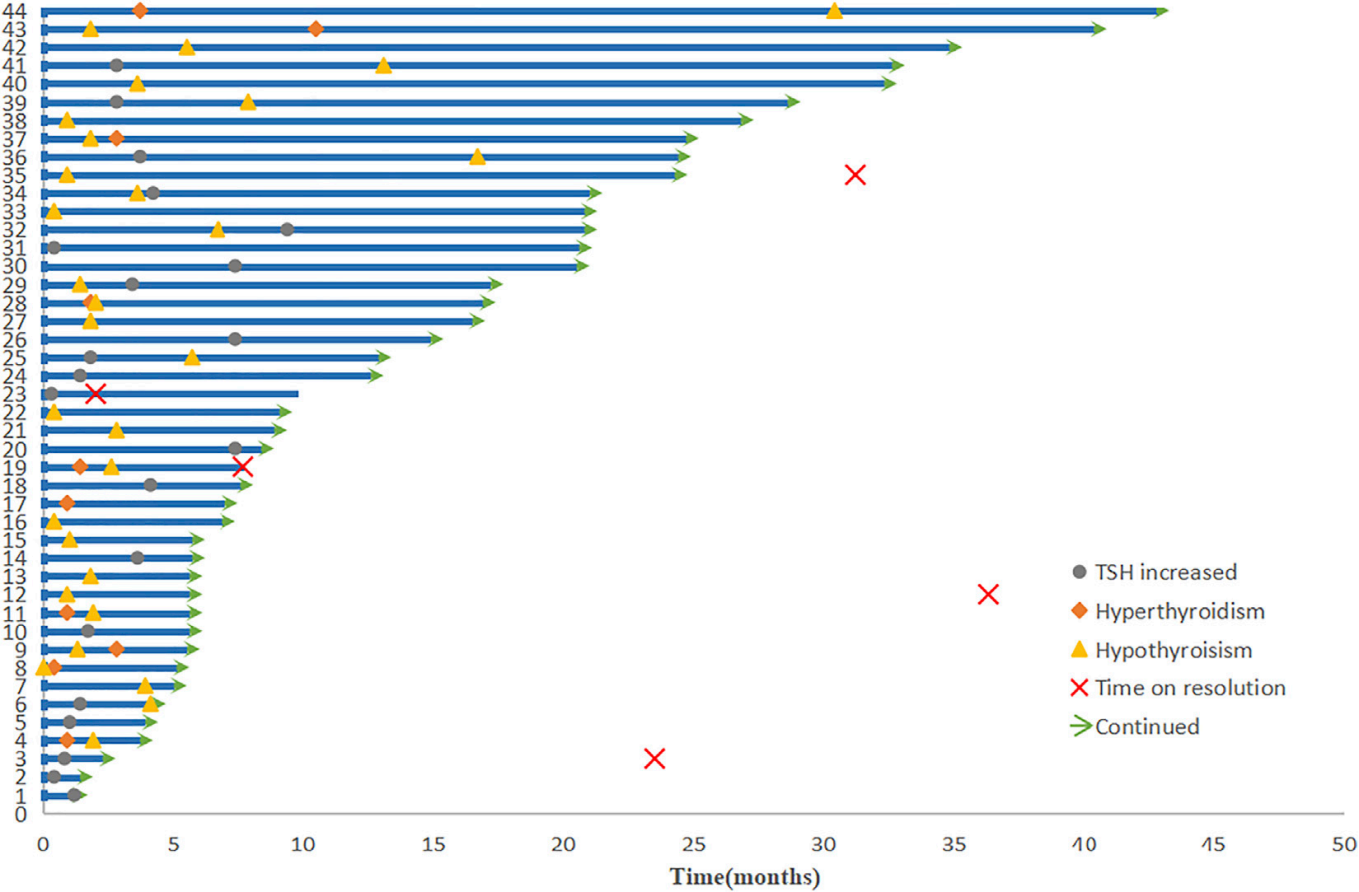
Duration of treatment and onset and resolution of endocrine dysfunctions.

The most frequent immune-related AE (irAEs) endocrine dysfunctions in descending order are hypothyroidism (*n* = 30), hyperglycaemia (*n* = 19), and hyperthyroidism (*n* = 10). The median time of endocrine dysfunctions was 6.7 weeks (range, 1.3–40.9 weeks). Thyroid dysfunction was a common AE and included both hypo- and hyperthyroidism. Among the patients with thyroid dysfunction, nine experienced hypothyroidism followed by hyperthyroidism, 21 had only hypothyroidism, 13 had only high levels of thyroid-stimulating hormone (TSH), and 2 had only hyperthyroidism. The median time to hypothyroidism after the onset of hyperthyroidism was 11.1 weeks. Of the patients with hypothyroidism, only five had it resolved completely with time; other patients reached clinical remission by medical control (Figure 3).

The other most common irAEs included increased amylase levels (*n* = 12), vitiligo (*n* = 9), hepatitis (*n* = 4), myositis (*n* = 1), and pneumonitis (*n* = 1). We found no instances of myocarditis or colitis. Abnormal liver function tests and diarrhoea are considered irAEs during anti–PD-1 antibody monotherapy; distinguishing between immune-related and antiangiogenic therapy–related AEs is difficult during combined therapy. Five patients had to receive systemic corticosteroids to manage their TRAEs. Among them, three had hepatic AEs, one had myositis and hepatotoxicity at the same time, and one had renal AEs. The median time from the onset of AEs to the administration of corticosteroids was 1 week, and the median corticosteroid treatment period was 8 weeks. One of these patients had a persistent AE until the end of the corticosteroid treatment.

Many patients in our cohorts exhibited hypertension, hand-foot syndrome, oral mucositis, hoarseness, cardiac effect, and proteinuria that correlated with antiangiogenic therapy. Nearly half of these patients had proteinuria (*n* = 34), followed by those with hand-foot syndrome (*n* = 31), hypertension (*n* = 21), arrhythmia (*n* = 15), and oral mucositis (*n* = 14).

### Safety Profile of Different Regimens

In our study, patients were treated with one of two combination therapy regimens: 30 patients (41.7%) were treated with camrelizumab combined with apatinib, and 42 patients (58.3%) were treated with toripalimab combined with axitinib. TRAEs were reported in 30 patients treated with camrelizumab plus apatinib and in 40 treated with toripalimab plus axitinib (Table 2). The camrelizumab plus apatinib regimen led to more grade ≥3 TRAEs than the toripalimab plus axitinib regimen (most AEs consisted in abnormal liver function test results). Similarly, hepatic AEs leading to treatment discontinuation were more common in the patients receiving the camrelizumab plus apatinib regimen. The patients treated with toripalimab plus axitinib, who had to interrupt their treatment due to AEs, displayed a wide range of symptoms (Figure 2; Supplementary Table S2).

**FIGURE 4:**
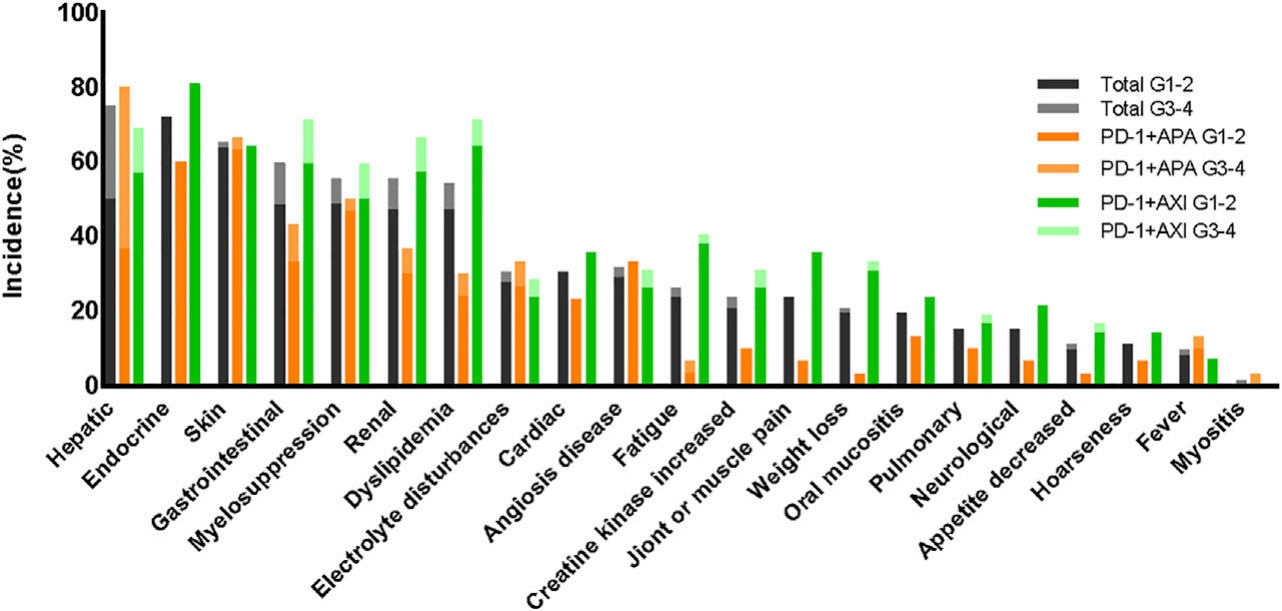
Treatment-related adverse events between different therapeutic regimens.

The AE spectra were similar between the regimens (Figure 4). Supplementary Table S2 lists the TRAEs experienced by >10% of patients. The incidences of irAEs were similar between regimens. Of note, all hepatitis (*n* = 4) or reactive cutaneous capillary endothelial proliferation (RCCEP) (*n* = 3) cases occurred in the patients receiving the camrelizumab plus apatinib regimen. In addition, there was a higher incidence of endocrine dysfunctions and gastrointestinal reactions in those receiving the toripalimab plus axitinib regimen. The incidences of proteinuria, hypertension, and oral mucositis, which are generally associated with antiangiogenic therapy, were more common in the patients receiving the toripalimab plus axitinib regimen.

### Impact of AEs on Response Rates and PFS

**FIGURE 5:**
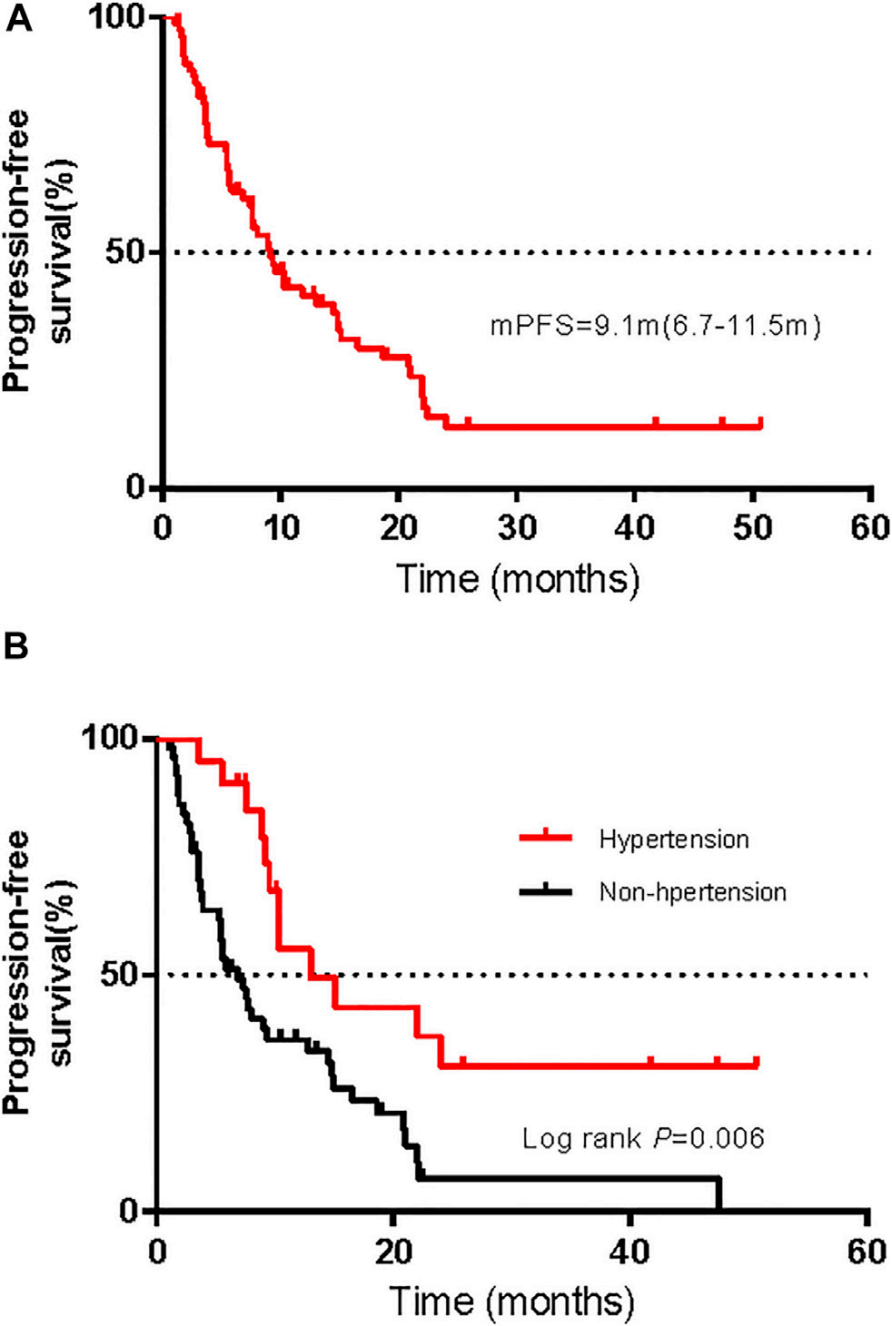
Kaplan–Meier curves of progression-free survival (PFS) for all patients **(A)** and comparison of PFS in patients with and without hypertension **(B)**.

Among the 72 patients in the whole cohort, the ORR was 36.1% (95% CI, 25.0–47.2%) and the median PFS was 9.1 months (95% CI, 6.7–11.5 months); see Figure 5A. Using univariate logistic regression models identified hypertension, renal toxicity, vitiligo, and endocrine dysfunction as factors associated with the ORR of advanced melanoma. After adjusting for pathological types, AJCC stages, hypertension, renal toxicity, vitiligo, and endocrine dysfunction, our multivariable logistic regression models showed that treatment-related AEs were not associated with the ORR (Supplementary Tables S3, S4). Moreover, univariable Cox regression analysis displayed a correlation between hepatic AEs, gastrointestinal AEs, cardiotoxicity, hypertension, and endocrine dysfunction with the PFS. Multivariate Cox proportional risk models that were carried out showed an association between the presence of hypertension and a long PFS, while other AEs were not associated with the PFS (Supplementary Tables S5, S6). Figure 5B shows the Kaplan–Meier PFS curves for patients with or without hypertension.

## Discussion

VEGF activation induces angiogenesis, an important characteristic of tumors (Carmeliet and Jain, 2011). Furthermore, the VEGF expression level has been associated with poor prognosis in patients with mucosal melanoma (Akiyama et al., 2018). Hence, VEGFR has become a treatment target in patients with different tumor types (liver cancer, renal carcinoma, etc.) (Kudo et al., 2018; Hao and Wang, 2020). Apatinib and axitinib are small molecule tyrosine kinase inhibitors (TKIs) of different VEGFR actions. Regimens of anti–PD-L/PD-L1 combined with antiangiogenic therapy have been efficient against a wide variety of solid tumors (Khan and Kerbel, 2018), such as endometrial cancer (Makker et al., 2020) and renal carcinoma (Taylor et al., 2020). Anti–PD-1 combined with antiangiogenic therapy has shown promising antitumor activity (Sheng et al., 2019). In this study, we characterized the safety profile of anti–PD-1 combined with antiangiogenic therapy. To the best of our knowledge, this is the first study to catalogue the TRAEs of anti–PD-1 plus antiangiogenic therapy in patients with noncutaneous melanoma.

We found that almost all of the patients experienced TRAEs, but most TRAEs were mild. Nearly half of the patients experienced grade ≥3 AEs. Grade 4 AEs occurred in five patients, and serious AEs occurred in <13% of patients. The incidences of all-grade AEs were consistent with those observed in other combination therapy trials, without unexpected AEs. A phase IB/II trial of 137 patients with different advanced solid tumors, including 22 patients with advanced melanoma, reported all-grade TRAEs in 97% of patients treated with lenvatinib plus pembrolizumab and grade 3 or 4 TRAEs in 33 and 2% of patients, respectively (Taylor et al., 2020). TRAE spectra differ between patients receiving immunology as monotherapy or combined with ipilimumab (anti–CTLA-4); however, the immune-related AEs were similar to those after anti–PD-1/PD-L1 with or without ipilimumab in patients with advanced melanoma (Robert et al., 2015; Wolchok et al., 2015). The time of onset of TRAEs after the start of treatment was also similar to that after initiating anti–PD-1/PD-L1 monotherapy (within 1–2 months) (Weber et al., 2017a; Martins et al., 2019). Renal AEs were the earliest AEs (median time to onset, 1.7 weeks) and cardiac AEs were the most delayed AEs (median time to onset, 20 weeks). Most TRAEs appeared within 1–4 months, and electrolyte disturbances had the shortest time to resolution (median, 2.7 weeks). However, the prevalences of irAEs differed. In our study, only five patients received corticosteroids to manage TRAEs; we did not analyze the impact of corticosteroids on antitumor efficacy. Another study has shown that corticosteroids do not affect antitumor responses (Weber et al., 2017a).

Hepatic AEs were the most common AEs and occurred in 54 patients. These AEs consisted mostly in abnormal liver function tests (increased ALT levels in 50%, increased AST levels in 33.3%), hyperbilirubinemia in 44.4%, and increased GGT levels in 8.6%. Of these 54 patients, four developed hepatitis. The rate of all-grade hepatotoxicity observed in this study was markedly increased compared with the rate for anti–PD-1/-PD-L1 monotherapy (3–9%) (Suzman et al., 2018) or antiangiogenic monotherapy (approximately 50%) (Hutson et al., 2013). Overlapping toxicities may be responsible for this difference.

Endocrine dysfunctions graded 1–2 were the second most common TRAEs in our study; they included hypothyroidism (41.7%), hyperglycaemia (26.4%), and hyperthyroidism (13.9%). A meta-analysis to compare the prevalences of endocrine dysfunctions with different immunotherapy regimens involving 19,922 patients found that the incidence of hypothyroidism was higher in patients treated with anti–PD-1 monotherapy (nearly 5–8.0%) than in patients treated with ipilimumab monotherapy (3.8%) (de Filette et al., 2019). Clinical trials have reported an incidence of hypothyroidism of 21% in patients receiving axitinib monotherapy. We found eight patients diagnosed with hypothyroidism followed by hyperthyroidism, and the hypothyroidism in most patients never got resolved and they needed permanent hormone replacement therapy. This coincides with previous findings (Lee et al., 2017).

Dermatologic toxicity is the most frequently reported irAE in patients who receive anti–PD-1/PD-L1 monotherapy (Villadolid and Amin, 2015; Kumar et al., 2017), and nearly one-third of patients experience dermatologic AEs including rash, pruritus, and vitiligo. In our study, skin AEs occurred in 64.5% of patients; the incidences of rash, pruritus, and vitiligo are similar to those after anti–PD-1 monotherapy (10–20%) (Weber et al., 2017b; Eggermont et al., 2018). By contrast, our cohort presented a high incidence of hand-foot syndrome (43.1%), a common AE in patients treated with TKIs. A meta-analysis including 57 studies and 24,956 patients showed the incidence of the all-grade hand-foot syndrome at 35% in patients who received VEGFR-TKIs (Ding et al., 2020). RCCEP (on the surface of the skin) is the most common AE related to camrelizumab. In the previous study, 66.8% of patients treated with camrelizumab experienced RCCEP (Wang et al., 2020). In contrast, in our study, the incidence of RCCEP was low. Apatinib may be a factor contributing to this difference.

Diarrhoea is the most frequently gastrointestinal irAE in patients receiving ipilimumab (34%), a frequency higher than that after anti–PD-1 monotherapy (21%) (Wolchok et al., 2017). Similarly, diarrhoea is also the most widely reported gastrointestinal toxicity (approximately 50%) in patients receiving TKI monotherapy (Kudo et al., 2018). Despite these overlapping toxicities, the incidence of diarrhoea was not significantly increased in our study.

Myelosuppression is the most widely reported AE in patients receiving chemotherapy. However, it was rare in the first immunotherapy clinical trials. More than half of the patients in our study experienced transient, reversible myelosuppression (leukopenia, neutropenia, thrombocytopenia, and anaemia) that was responsive to growth factors and was usually resolved within weeks (median 2.4 weeks); only a few patients developed persistent anaemia or leukopenia. Ethnicity may influence the tolerance of therapy. In a clinical study of 36 Chinese patients, nearly one-third experienced anaemia, and a quarter experienced leukopenia (Tang et al., 2019).

A meta-analysis comprising more than 16,000,000 adverse drug reactions and involving 613 fatal irAEs found that anti–PD-1/PD-L1 fatalities were commonly due to pneumonitis (35%), hepatitis (22%), neurotoxic effects (15%), cardiac effects (8%), and myositis (7%) (Wang et al., 2018). In this study, we did not observe TRAEs leading to death. In addition, all cardiac or neurological TRAEs were grade 1 or 2. Pneumonitis and myositis occurred in one patient each.

Hypertension, hand-foot syndrome, oral mucositis, hoarseness, fistula formation, and proteinuria are associated with antiangiogenic therapy. These toxicities are associated with the antitumor mechanism of VEGFR inhibitors. The rates of hypertension and proteinuria in previous clinical trials with lenvatinib plus pembrolizumab were approximately 27% and 23%, respectively, in patients with advanced renal cell cancer (Hutson et al., 2013). As mentioned above, the AEs in our study were similar to those in other combination therapy clinical trials. However, the incidence of TRAEs was higher than those in previous studies. In our study, one patient developed oesophageal fistula. Fistula formation is a rare event that usually occurs in bevacizumab-treated patients (Ostby et al., 2020). The causes of this difference include the overlapping toxicities and the different tumor types between studies. In our study, all patients had non-cutaneous melanoma; the patients in most other relevant studies had other solid tumors, such as renal cell tumor and liver cancer.

Apatinib and axitinib are both orally bioavailable small-molecule antiangiogenic agents and inhibit the tyrosine kinase activity of VEGFR, resulting in tumor angiogenesis inhibition. The VEGFR family comprises three receptor tyrosine kinases (TKRs), namely, VEGFR-1, VEGFR-2, and VEGFR-3, and VEGFR-2 is the main signaling TKR (Ferrara and Kerbel, 2005). Apatinib selectively inhibits VEGFR-2 (Tian et al., 2011). Of note, axitinib is a multitarget agent inhibiting VEGFR 1-3, c-KIT, and PDGF receptors (Hu-Lowe et al., 2008). In this study, the incidences of proteinuria, hypertension, and oral mucositis (AEs usually associated with antiangiogenic therapy in studies on camrelizumab combined with apatinib) were higher in patients receiving the toripalimab plus axitinib regimen. The difference of antitumor mechanisms between apatinib and axitinib might be the reason for this discrepancy. Some studies have positively associated clinical outcomes with the presence of vitiligo and hypertension (Quach et al., 2019), but others have shown opposing results (Ascierto et al., 2014). In our study, we found that hypertension was positively associated with PFS.

We are aware of the limitations in our study. First, all data were derived from prospective clinical trials at our centre, which ensures the integrity of the data, but excluded patients with a history of autoimmune diseases, ongoing infections, organ dysfunction, and others. Future studies should determine the spectrum of toxic effects of combined therapy in the real world. Second, although we collected all the long-term survival data available, most patients with mild AEs were lost to follow-up AE’s outcomes after the end of treatment. Thus, our time to resolution may be inaccurate due to missing information. Finally, although this study is the largest to date on the safety profile of anti–PD-1 combined with TKIs in patients with acral and mucosal melanoma, our sample size was small. Larger sample sizes and longer follow-ups are needed to determine the long-term safety profile of these therapies.

## Data Availability

The raw data supporting the conclusions of this article will be made available by the authors, without undue reservation.
