# Supplementary material for: Safety Profile of Immunotherapy Combined With Antiangiogenic Therapy in Patients With Melanoma: Analysis of Three Clinical Studies
Source: Front Pharmacol. 2021 Nov 9;12:747416. doi: 10.3389/fphar.2021.747416 (PMC8630676; doi:10.3389/fphar.2021.747416)
Supplement: Supplementary file 1 [file Table1.docx]

Supplementary Material

# Supplementary Data

Not applicable.

# Supplementary Tables

**eTable 1 Treatment-related adverse events(＞10%) leading to discontinuation**

| Adverse Event | N=72 | | APA+PD-1 N=30 | | AXI+PD-1 N=42 | |
| --- | --- | --- | --- | --- | --- | --- |
|  | Any Grade | Grade 3 or 4 | Any Grade | Grade 3 or 4 | Any Grade | Grade 3 or 4 |
| TRAEs leading to discontinuation | 32(44.4) | 26(36.1) | 15(50.0) | 11(36.7) | 18(42.9) | 15(35.7) |
| Hepatic | 19(26.4) | 12(16.7) | 15(50.0) | 9(30.0) | 4(9.5) | 3(7.1) |
| ALT increased | 9(12.5) | 0 | 6(20.0) | 5(16.7) | 3(7.1) | 2(4.8) |
| AST increased | 6(8.3) | 4(5.6) | 5(16.7) | 3(10.0) | 1(2.4) | 1(2.4) |
| Hepatitis | 4(5.6) | 4(5.6) | 4(13.3) | 4(13.3) | 0 | 0 |
| Hyperbilirubinemia | 1(1.4) | 1(1.4) | 0 | 0 | 1(2.4) | 1(2.4) |
| Gastrointestinal | 7(9.7) | 4(5.6) | 1(3.3) | 1(3.3) | 6(14.3) | 3(7.1) |
| Diarrhea | 3(4.2) | 1(1.4) | 0 | 0 | 3(7.1) | 1(2.4) |
| Bloody stool | 1(1.4) | 0 | 0 | 0 | 1(2.4) | 0 |
| Vomiting | 1(1.4) | 1(1.4) | 1(3.3) | 1(3.3) | 0 | 0 |
| Intestinal obstruction | 1(1.4) | 0 | 0 | 0 | 1(2.4) | 0 |
| Esophageal fistula | 1(1.4) | 1(1.4) | 0 | 0 | 1(2.4) | 1(2.4) |
| Amylase increased | 1(1.4) | 1(1.4) | 0 | 0 | **1(2.4)** | **1(2.4)** |
| Renal | 6(8.3) | 5(6.9) | 1(3.3) | 1(3.3) | 5(11.9) | 4(9.5) |
| Proteinuria | 5(6.9) | 4(5.6) | 1(3.3) | 1(3.3) | 4(9.5) | 3(7.1) |
| Blood creatinine increased | 1(1.4) | 1(1.4) | 0 | 0 | 1(2.4) | 1(2.4) |
| Skin | 4(5.6) | 1(1.4) | 2(6.7) | 1(3.3) | 2(4.8) | 0 |
| Wound nonhealing | 2(2.8) | 0 | 1(3.3) | 0 | 1(2.4) | 0 |
| Rash | 1(1.4) | 0 | 0 | 0 | 1(2.4) | 0 |
| Wound infection | 1(1.4) | 1(1.4) | 1(3.3) | 1(3.3) | 0 | 0 |
| Myelosuppression | 4(5.6) | 2(2.8) | 1(3.3) | 0 | 3(7.1) | 2(4.8) |
| Anaemia | 2(2.8) | 1(1.4) | 0 | 0 | 2(4.8) | 1(2.4) |
| Neutrocytopenia | 1(1.4) | 1(1.4) | 0 | 0 | 1(2.4) | 1(2.4) |
| Thrombopenia | 1(1.4) | 0 | 1(3.3) | 0 | 0 | 0 |
| High triglyceride | 3(4.2) | 2(2.8) | 0 | 0 | 3(7.1) | 2(4.8) |
| Electrolyte disturbances | 2(2.8) | 2(2.8) | 0 | 0 | 2(4.8) | 2(4.8) |
| Hypokalemia | 1(1.4) | 1(1.4) | 0 | 0 | 1(2.4) | 1(2.4) |
| Hyponatremia | 1(1.4) | 1(1.4) | 0 | 0 | 1(2.4) | 1(2.4) |
| Creatine kinase increased | 2(2.8) | 2(2.8) | 0 | 0 | 2(4.8) | 2(4.8) |
| Fatigue | 1(1.4) | 1(1.4) | 0 | 0 | 1(2.4) | 1(2.4) |
| Weight loss | 1(1.4) | 0 | 0 | 0 | 1(2.4) | 0 |
| Hypertension | 1(1.4) | 1(1.4) | 0 | 0 | 1(2.4) | 1(2.4) |
| Myositis | 1(1.4) | 1(1.4) | 1(3.3) | 1(3.3) | 0 | 0 |
| Eyelid edema | 1(1.4) | 0 | 0 | 0 | 1(2.4) | 0 |

**eTable 2 Any treatment-related AE in >10% of patients**

| Adverse Event,n(%) | N=72 | | PD-1+APA N=30 | | PD-1+AXI N=42 | |
| --- | --- | --- | --- | --- | --- | --- |
|  | Any Grade | Grade 3 or 4 | Any Grade | Grade 3 or 4 | Any Grade | Grade 3 or 4 |
| Hepatic | 54(75.0) | 18(25.0) | 24(80.0) | 13(43.3) | 29(69.0) | 5(11.9) |
| ALT increased | 36(50.0) | 12(16.7) | 16(53.3) | 10(33.3) | 20(47.6) | 2(4.8) |
| Hyperbilirubinemia | 32(44.4) | 1(1.4) | 17(56.7) | 0 | 15(35.7) | 1(2.4) |
| AST increased | 24(33.3) | 4(5.6) | 12(40.0) | 3(10.0) | 12(28.6) | 1(2.4) |
| GGT increased | 6(8.3) | 3(4.2) | 1(3.3) | 1(3.3) | 5(11.9) | 2(4.8) |
| Hepatitis | 4(5.6) | 4(5.6) | 4(13.3) | 4(13.3) | 0 | 0 |
| Endocrine | 52(72.2) | 0 | 18(60.0) | 0 | 34(81.0) | 0 |
| Hypothyroidism | 30(41.7) | 0 | 8(26.7) | 0 | 22(52.4) | 0 |
| Hyperglycemia | 19(26.4) | 0 | 5(16.7) | 0 | 17(40.5) | 0 |
| Hyperthyroidism | 10(13.9) | 0 | 3(4.2) | 0 | 7(16.7) | 0 |
| Skin | 47(65.3) | 1(1.4) | 20(66.7) | 1(3.3) | 27(64.3) | 0 |
| Hand-food syndrome | 31(43.1) | 0 | 13(43.3) | 0 | 18(42.9) | 0 |
| Rash | 22(30.6) | 0 | 9(30.0) | 0 | 13(31.0) | 0 |
| Vitiligo | 9(12.5) | 0 | 4(13.3) | 0 | 5(11.9) | 0 |
| Pruritus | 4(5.6) | 0 | 0 | 0 | 4(9.5) | 0 |
| Eczema | 4(5.6) | 0 | 2(6.7) | 0 | 2(4.8) | 0 |
| Alopecia | 4(5.6) | 0 | 1(3.3) | 0 | 3(7.1) | 0 |
| Wound nonhealing | 3(4.2) | 0 | 2(6.7) | 0 | 1(2.4) | 0 |
| RCCEP | 3(4.2) | 0 | 3(10.0) | 0 | 0 | 0 |
| Wound infection | 1(1.4) | 1(1.4) | 1(3.3) | 1(3.3) | 0 | 0 |
| Gastrointestinal | 43(59.7) | 8(11.1) | 13(43.3) | 3(10.0) | 30(71.4) | 5(11.9) |
| Diarrhea | 27(37.5) | 4(5.6) | 6(20.0) | 1(3.3) | 21(50.0) | 3(7.1) |
| Nausea | 13(18.1) | 0 | 5(16.7) | 0 | 8(19.0) | 0 |
| Abdominal pain | 12(16.7) | 0 | 1(3.3) | 0 | 11(26.2) | 0 |
| Amylase increased | 12(16.7) | 0 | 1(3.3) | 0 | 11(26.2) | 1(2.4) |
| Appetite decreased | 10(13.9) | 1(1.4) | 3(10.0) | 0 | 7(16.7) | 1(2.4) |
| Vomiting | 7(9.7) | 1(1.4) | 5(16.7) | 1(3.3) | 2(4.8) | 0 |
| Esophageal fistula | 1(1.4) | 1(1.4) | 0 | 0 | 1(2.4) | 1(2.4) |
| Myelosuppression | 40(55.6) | 5(6.9) | 15(50.0) | 1(3.3) | 25(59.5) | 4(9.5) |
| Leukopenia | 26(36.1) | 1(1.4) | 10(33.3) | 0 | 13(31.0) | 1(2.4) |
| Neutrocytopenia | 15(20.8) | 4(5.6) | 4(13.3) | 1(3.3) | 11(26.2) | 3(7.1) |
| Thrombopenia | 12(16.7) | 0 | 7(23.3) | 0 | 5(11.9) | 0 |
| Anaemia | 11(15.3) | 2(2.8) | 0 | 0 | 11(26.2) | 2(4.8) |
| Renal | 40(55.6) | 6(8.3) | 11(36.7) | 2(6.7) | 28(66.7) | 4(9.5) |
| Proteinuria | 34(47.2) | 5(6.9) | 11(36.7) | 2(6.7) | 23(54.8) | 3(7.1) |
| Leukocyturia | 8(11.1) | 0 | 1(3.3) | 0 | 8(19.0) | 0 |
| Hematuresis | 8(11.1) | 0 | 3(10.0) | 0 | 5(11.9) | 0 |
| Blood creatinine increased | 3(4.2) | 1(1.4) | 0 | 0 | 3(7.1) | 1(2.4) |
| Urinary tract irritation symptoms | 3(4.2) | 0 | 0 | 0 | 3(7.1) | 0 |
| Dyslipidemia | 39(54.2) | 5(6.9) | 9(30.0) | 2(6.7) | 30(71.4) | 3(7.1) |
| High cholesterol | 28(38.9) | 0 | 6(20.0) | 0 | 22(52.4) | 0 |
| High triglyceride | 27(37.5) | 5(6.9) | 6(20.0) | 2(6.7) | 21(50.0) | 3(7.1) |
| Electrolyte disturbances | 22(30.6) | 2(2.8) | 10(33.3) | 0 | 12(28.6) | 2(4.8) |
| Hypokalemia | 17(23.6) | 0 | 8(26.7) | 0 | 9(21.4) | 2(4.8) |
| Cardiac | 22(30.6) | 0 | 7(23.3) | 0 | 15(35.7) | 0 |
| Arhythmia | 15(20.8) | 0 | 5(16.7) | 0 | 10(23.8) | 0 |
| Abnormal ECG | 12(16.7) | 0 | 4(13.3) | 0 | 8(19.0) | 0 |
| Hypertension | 21(29.2) | 2(2.8) | 8(26.7) | 0 | 13(31.0) | 2(4.8) |
| Fatigue | 19(26.4) | 2(2.8) | 2(6.7) | 1(3.3) | 17(40.5) | 1(2.4) |
| Creatine kinase increased | 17(23.6) | 2(2.8) | 3(10.0) | 0 | 13(31.0) | 2(4.8) |
| Jiont or muscle pain | 17(23.6) | 0 | 2(6.7) | 0 | 15(35.7) | 0 |
| Weight loss | 15(20.8) | 1(1.4) | 1(3.3) | 0 | 14(33.3) | 1(2.4) |
| Oral mucositis | 14(19.4) | 0 | 4(13.3) | 0 | 10(23.8) | 0 |
| Total uric acid increased | 14(19.4) | 0 | 1(3.3) | 0 | 13(31.0) | 0 |
| Pulmonary | 11(15.3) | 0 | 3(10.0) | 0 | 8(19.0) | 1(2.4) |
| Cough | 7(9.7) | 0 | 0 | 0 | 7(16.7) | 0 |
| Upper respiratory infection | 6(8.3) | 0 | 3(10.0) | 0 | 3(7.1) | 0 |
| Pneumonitis | 1(1.4) | 1(1.4) | 0 | 0 | 1(2.4) | 1(2.4) |
| Neurological | 11(15.3) | 0 | 2(6.7) | 0 | 9(21.4) | 0 |
| Numbness | 3(4.2) | 0 | 0 | 0 | 3(7.1) | 0 |
| Insomnia | 3(4.2) | 0 | 0 | 0 | 3(7.1) | 0 |
| Headache | 9(12.5) | 0 | 2(6.7) | 0 | 7(16.7) | 0 |
| Dizzy | 2(2.8) | 0 | 0 | 0 | 2(4.8) | 0 |
| Appetite decreased | 8(11.1) | 1(1.4) | 1(3.3) | 0 | 7(16.7) | 1(2.4) |
| Hoarseness | 8(11.1) | 0 | 2(6.7) | 0 | 6(14.3) | 0 |
| Fever | 7(9.7) | 1(1.4) | 4(13.3) | 1(3.3) | 3(7.1) | 0 |
| Myositis | 1(1.4) | 1(1.4) | 1(3.3) | 1(3.3) | 0 | 0 |

RCCEP, Reactive cutaneous capillary endothelial proliferation.

eTable 3 Univariate logistic regression models of risk factors for ORR in patients with melanoma treated with immunotherapy combined with antiangiogenic therapy

| Characteristics |  | P value | RR(95%CI) |
| --- | --- | --- | --- |
| Age, years |  | 0.52 | 0.99(0.94-1.03) |
| Sex,N(%) | Female | reference |  |
|  | Male | 0.71 | 0.83(0.32-2.2) |
| Pathological type,N(%) | Mucosal | reference |  |
|  | Acral | 0.12 | 2.25(0.81-6.25) |
| Mutations status,N(%) | wild type | reference |  |
|  | mutated | 0.71 | 0.79(0.22-2.78) |
|  | UK | 0.19 | 0.47(0.15-1.45) |
| LDH,N(%) | ≤ULN | reference |  |
|  | ＞ULN | 0.51 | 1.42(0.51-3.97) |
| ECOG,N(%) | 0 | reference |  |
|  | 1 | 0.2 | 0.51(0.18-1.42) |
| AJCC stage,N(%) | III | reference |  |
|  | IV | 0.13 | 2.38(0.78-7.25) |
| Prior systemic therapy,N(%) | No | reference |  |
|  | Yes | 0.84 | 0.87(0.22-3.42) |
| Hepatic | No | reference |  |
|  | Yes | 0.86 | 0.9(0.29-2.81) |
| Gastrointestinal | No | reference |  |
|  | Yes | 0.62 | 1.28(0.48-3.46) |
| Renal | No | reference |  |
|  | Yes | 0.03 | 0.31(0.11-0.88) |
| Skin | No | reference |  |
|  | Yes | 0.14 | 2.2(0.78-6.19) |
| Myelosuppression | No | reference |  |
|  | Yes | 0.16 | 0.48(0.18-1.32) |
| Hypertension | No | reference |  |
|  | Yes | 0.03 | 0.3(0.1-0.87) |
| Cardiac | No | reference |  |
|  | Yes | 0.16 | 0.48(0.18-1.32) |
| Pulmonary | No | reference |  |
|  | Yes | 0.62 | 1.28(0.48-3.46) |
| Vitiligo | No | reference |  |
|  | Yes | 0.13 | 0.31(0.07-1.41) |
| Endocrine | No | reference |  |
|  | Yes | 0.02 | 0.15(0.03-0.7) |

eTable 4 Multivariate logistic regression models of risk factors for ORR in patients with melanoma treated with immunotherapy combined with antiangiogenic therapy

| Characteristics |  | P value | OR(95%CI) |
| --- | --- | --- | --- |
| Pathological type,N(%) | Mucosal | reference |  |
|  | Acral | 0.24 | 2.1(0.61-7.18) |
| AJCC stage,N(%) | III | reference |  |
|  | IV | 0.09 | 3.07(0.83-11.32) |
| Renal | No | reference |  |
|  | Yes | 0.53 | 0.65(0.17-2.51) |
| Hypertension | No | reference |  |
|  | Yes | 0.06 | 0.3(0.09-1.04) |
| Vitiligo | No | reference |  |
|  | Yes | 0.07 | 0.16(0.02-1.17) |
| Endocrine | No | reference |  |
|  | Yes | 0.14 | 0.22(0.03-1.61) |

eTable 5 Univariate Cox regression models of PFS in patients with melanoma treated with immunotherapy combined with antiangiogenic therapy

| Characteristics |  | P value | HR(95%CI) |
| --- | --- | --- | --- |
| Age, years |  | 0.81 | 1(0.97-1.02) |
| Sex,N(%) | Female | reference |  |
|  | Male | 0.72 | 0.91(0.53-1.56) |
| Pathological type,N(%) | Mucosal | reference |  |
|  | Acral | 0.35 | 0.77(0.44-1.34) |
| Mutations status,N(%) | wild type | reference |  |
|  | mutated | 0.57 | 0.82(0.41-1.64) |
|  | UK | 0.59 | 0.85(0.46-1.57) |
| LDH,N(%) | ≤ULN | reference |  |
|  | ＞ULN | 0.01 | 2.2(1.25-3.86) |
| ECOG,N(%) | 0 | reference |  |
|  | 1 | 0.57 | 1.18(0.67-2.06) |
| AJCC stage,N(%) | III | reference |  |
|  | IV | 0.31 | 1.41(0.73-2.73) |
| Prior systemic therapy,N(%) | No | reference |  |
|  | Yes | 0.71 | 0.87(0.42-1.8) |
| Hepatic | No | reference |  |
|  | Yes | 0.06 | 0.56(0.3-1.04) |
| Gastrointestinal | No | reference |  |
|  | Yes | 0.03 | 0.48(0.26-0.92) |
| Renal | No | reference |  |
|  | Yes | 0.12 | 0.64(0.37-1.13) |
| Skin | No | reference |  |
|  | Yes | 0.89 | 0.96(0.53-1.73) |
| Myelosuppression | No | reference |  |
|  | Yes | 0.11 | 0.64(0.37-1.1) |
| Hypertension | No | reference |  |
|  | Yes | 0.01 | 0.39(0.2-0.77) |
| Cardiac | No | reference |  |
|  | Yes | 0.08 | 0.59(0.32-1.07) |
| Pulmonary | No | reference |  |
|  | Yes | 0.82 | 1.09(0.53-2.24) |
| Vitiligo | No | reference |  |
|  | Yes | 0.14 | 0.5(0.2-1.26) |
| Endocrine | No | reference |  |
|  | Yes | 0.03 | 0.48(0.26-0.92) |

eTable 6 Multivariate Cox regression models of PFS in patients with melanoma treated with immunotherapy combined with antiangiogenic therapy

| Characteristics |  | P value | HR(95%CI) |
| --- | --- | --- | --- |
| Pathological type,N(%) | Mucosal | reference |  |
|  | Acral | 0.43 | 0.76(0.38-1.5) |
| AJCC stage,N(%) | III | reference |  |
|  | IV | 0.2 | 1.66(0.77-3.6) |
| LDH,N(%) | ≤ULN | reference |  |
|  | ＞ULN | 0.1 | 1.64(0.9-2.98) |
| Hepatic | No | reference |  |
|  | Yes | 0.2 | 0.56(0.23-1.37) |
| Gastrointestinal | No | reference |  |
|  | Yes | 0.27 | 0.67(0.32-1.37) |
| Hypertension | No | reference |  |
|  | Yes | 0.01 | 0.39(0.2-0.79) |
| Cardiac | No | reference |  |
|  | Yes | 0.57 | 0.83(0.43-1.59) |
| Endocrine | No | reference |  |
|  | Yes | 0.83 | 0.9(0.37-2.23) |

# Supplementary Figures

Not applicable.
